# A case report of dengue haemorrhagic fever during the peripartum period: challenges in management and a case of vertical dengue transmission

**DOI:** 10.1186/s12879-018-3352-x

**Published:** 2018-08-28

**Authors:** B. V. K. M. Bopeththa, Sardha Hemapriya, K. K. Gayan Niranga, Danushka S. K. Kotigala

**Affiliations:** 1 Registrar in Emergency Medicine, Teaching Hospital Kandy, Kandy, Sri Lanka; 2 Consultant Obstetrician and Gynaecologist, Teaching Hospital Kandy, Kandy, Sri Lanka

**Keywords:** Dengue, Pregnancy, Vertical transmission, Physiological changes in pregnancy

## Abstract

**Background:**

Incidence of Dengue infection is on the increase in Sri Lanka with it being associated with significant maternal and neonatal morbidity in pregnancy.

**Case presentation:**

A 33-year-old pregnant woman at 38 weeks of gestation, presented with acute onset of fever, was later diagnosed with Dengue illness. Due to the emergence of warning symptoms and signs and rapidly dropping platelet count, the baby was delivered by urgent caesarian section. She went into the critical phase during the postoperative period and due to concealed bleeding, required blood transfusion. On the 5th day of life, the neonate was also diagnosed with possible dengue by vertical transmission and required blood and PLT transfusions for recovery.

**Conclusions:**

This case report illustrates how a high index of suspicion, early diagnosis, close monitoring, timely intervention and critical consideration of physiological changes of pregnancy when interpreting clinical situation, led to achieving a successful outcome.

## Background

Dengue fever is an *Aedes* mosquito-borne flavivirus infection. It is becoming endemic in Sri Lanka, mainly due to changes in the rainfall patterns that increase the incidence. Dengue has become a major public health issue in Sri Lanka causing 215 deaths up to now, from 2017 [1]. Dengue is commonly defined as an acute febrile illness, associated with two or more of the following signs or symptoms: an intense headache, retro-orbital pain, myalgia, arthralgia, skin rash, leucopenia and hemorrhagic manifestations [2]. Although management of dengue in pregnancy is similar to non-pregnant dengue patient management, due to various physiological changes occurring in pregnancy, it may cause significant diagnostic and management issues in practical clinical situations.

Dengue infection is associated with a number of maternal and neonatal complications including maternal death, a most severe form of miscarriages, preterm deliveries, fetal anomalies and neonatal deaths respectively [3, 4]. Although increasing number of vertical transmission of dengue fever cases have been reported, details of vertical transmission is lacking [5]. Here, we report a case of dengue hemorrhagic fever during peripartum period that includes a possible case of vertical transmission.

## Case presentation

A 33-year-old previously healthy mother in her 2nd pregnancy, was admitted at 38 weeks of gestation with a one-day history of high fever with chills, myalgia, arthralgia, and headache. Her first pregnancy ended up as lower segment caesarian section (LSCS) due to the unfavorable cervix. On admission, she was febrile (102 °F). Her pulse rate (PR) and blood pressure (BP) were 88 beats per minute and 94/60 mmHg respectively. Rest of the cardiac and respiratory examination was unremarkable. Abdominal examination revealed a soft abdomen with a single live fetus and symphysiofundal height was compatible with gestational age. Vaginal examination revealed an unfavorable cervix. Laboratory investigation results were as follows: total white cell count (WBC) 7100/mm^3^, platelet count (PLT) 112,000/mm^3^, haemoglobin (Hb) 11.5 g/dl, packed cell volume (PCV) 30%, C reactive protein (CRP) 31 mg/l and positive NS 1 antigen. Liver function and renal function tests were normal. Cardiotocograph (CTG) was normal and the fetal biophysical profile was compatible with the period of gestation (POG). There was no ultrasound evidence of free fluid in the abdomen or pelvis. The diagnosis was made of uncomplicated dengue and managed according to current national dengue management guidelines.

On the 2nd day, she developed two episodes of vomiting and had mild intermittent abdominal pain. Her PR was 98 beats per minute and blood pressure was 90/64 mmHg. There was mild right hypochondriac tenderness. Complete blood count (CBC) showed WBC 6900/mm^3^, PLT 72000mm^3^ and PCV 32%. Ultrasonically, there was no free in abdomen or chest. A multidisciplinary meeting was convened and a decision made to deliver her baby by urgent LSCS. Blood, fresh frozen plasma, and platelets were preserved and LSCS was performed under spinal anaesthesia. A healthy male baby weighing 3.0 kg was delivered with an uneventful intraoperative period. Although there was no significant bleeding as a prophylactic measure B lynch sutures were applied and an abdominal drain was also inserted. She was then transferred to intensive care unit (ICU) for further management.

On the 3rd day, fever was persisting and she continued to complain of right hypochondriac pain. She became tachycardic with PR of 105 beats per minute. BP was 92/66 mmHg. CBC revealed WBC of 5300/mm^3^ with PLT of 63,000/mm^3^ and PCV of 35%. Ultrasound (USS) examination showed pericholecystic oedema with a thin layer of free fluid in the hepatorenal pouch. The diagnosis of dengue hemorrhagic fever was made and observations conducted according to the observation chart for management of dengue in adult patients with fluid leakage. A 3 hourly PCV monitoring, hourly capillary refill time (CRFT), PR, supine BP and urine output was conducted. CBCs were performed twice daily with daily alanine transaminase (ALT) and aspartate transaminase (AST). Serum albumin was found to be low and 2D ECHO was normal. To cover unexpected nosocomial infections, IV of 4.5 g Piperazillin tazobactam, 8 hourly, was started. The summary of investigations is shown below (Table 1).

**Table 1:** Summary of investigations performed during critical phase

	Hours of critical phase	WBC (cells/mm^3^)	PLT (cells/mm^3^)	Hb (g/dl)	PCV (%)	AST (U/L)	ALT (U/L)	Blood sugar (Mg/dl)	Corrected Serum calcium (mmol/l)	Arterial PH
Day 01	1–12	10.5	27,000	11.2	34	17	08	104		7.43
	13–24	11.98	26,000	10.9	36	55	6	138		
Day 02	25–36	20.9	18,000	8.9	26	141	62	118	2.26	7.46
	37–48	17.6	36,000	10.2	32	122	34	142		

During the first 12 h of the critical phase (CP), vital parameters were stable and a flat rate of fluid administration was continued. Towards the peak of the leaking phase, her PR started to rise with a narrowing of pulse pressure. As there was a sudden drop in PCV, possible intra abdominal bleeding was suspected and 500 ml of packed cells was transfused. Although the abdominal draining was absent, USS confirmed the high possibility of the presence of blood in the peritoneal cavity. Haemodynamic parameters were improved following transfusion. At the same time, intravenous (IV) tranexamic acid 1 g was also given. Although there was a further drop in platelets, clotting studies were well within the normal range. At the beginning of the second half of the critical phase, she had developed pedal edema, mild ascites with right-sided mild pleural effusion without respiratory compromise. Around the 36th hour of CP, urine output dropped to less than 0.5 ml/kg/hour. Due to the high possibility of fluid overload, IV dextran 350 ml was given. Thereafter she had an uneventful recovery from rest of CP. She was then transferred to the high dependency unit and later to the postnatal ward, for further care. During the recovery phase, she became fever free and went into polyuric phase. Her CBC showed WBC 10000/mm^3^, PLT 238000/mm^3^ and Hb 9.8 g/dl. IV antibiotics were stopped after completion of the one week course following the negative urine and blood culture reports. Throughout the illness, her clotting studies and renal functions were normal. Later, she was discharged and underwent an uneventful recovery.

During her ICU stay, the baby was kept in the special baby unit (SBU) for the continuation of feeding and observation. He was never taken out of SBU and had no history of mosquito bites. The neonate was well until the 5th day of life but suddenly developed a high fever with jaundice. Sepsis or congenital dengue infection was suspected. Laboratory investigation results were as follows: WBC 5800/mm^3^, Hb 13.8 g/dl, PLT 126000/mm^3^, positive dengue NS 1 Antigen, AST 87 U/L, ALT 23 U/L, CRP 5.8 mg/l with normal liver and renal functions with the exception of an elevated indirect bilirubin of 35 micro mol/L. Diagnosis of dengue fever was made and managed according to current guidelines. Later, on the 7th day of life, his PLT count dropped from 86,000/mm^3^ to 43,000/mm^3^ and he developed malena. However, bedside USS revealed no evidence of fluid leakage or intracranial bleed. Haemodynamic parameters were within normal ranges except tachycardia of 170 bpm during the period of malena. 30 ml of PLT and 45 ml of packed red cell transfusion were done. With close monitoring and judicious fluid balance, the baby improved and was discharged from the SBU with PLT of 146,000/mm^3^ and Hb of 12 g/dl on the 11th day of life. However, his body weight had dropped to 2.580 kg upon discharge. On the 25th day of life, clinic review revealed weight gain up to 2.780 kg with age-appropriate milestone levels.

## Discussion and conclusions

Currently, dengue is the most significant mosquito-borne disease in Sri Lanka. It is transmitted by the bite of an *Aedes* mosquito infected with any one of the four dengue viruses. Laboratory results have found that Dengue virus serotype 2 (DENV-2) to be the circulating strain in this outbreak [1]. Due to heavy monsoon rains and improper waste disposal leading to stagnation of rainwater, is attributed to the increasing number of dengue cases.

The clinical assessment, diagnosis, treatment, and monitoring of dengue fever or dengue hemorrhagic fever during pregnancy can be very difficult due to various physiological changes occurring in pregnancy and other obstetric complications mimicking a clinical presentation of dengue infection. Fever with low PLT count, elevated liver enzymes, and right hypochondriac pain could be a part of HELLP syndrome or dengue illness. Labor pain can be misinterpreted as right upper quadrant pain. Therefore diagnosis of dengue can be misled by the presentation of other obstetric complications. Hence, clinicians should have a high index of suspicion for the diagnosis [3].

Having higher baseline heart rate and a fall in systolic and diastolic blood pressure induced by a reduction in systemic vascular resistance, may cause difficulty in early detection of entering the dengue critical phase, dengue shock syndrome and assessing response to fluid boluses as in this case.

Plasma volume expansion accompanied by a lesser increase in red cell volume causes a modest reduction in hematocrit [6]. Therefore it is very important to determine the baseline PCV during the early part of dengue illness, as non-pregnant PCV values cannot be applied to pregnant patients. Without a baseline PCV, it is difficult to recognize the objective evidence of plasma leakage or concealed bleeding. In our patient, concealed bleeding was recognized by the sudden drop of PCV. However, a 20% rise of baseline PCV at the entrance into critical phase was not evident in this case, probably due to satisfactory hydration matching the rate of leaking. Over hydration in dengue can cause edema, pleural effusion, ascites and respiratory distress. Pregnancy itself can cause edema and mild respiratory discomfort due to gravid uterus. Clinical judgment alone may not be sufficient in such situations as in this case.

Pregnancy is associated with leukocytosis, primarily related to the increased circulation of neutrophils. In addition, the platelet count also begins to rise soon after delivery. Dengue infection causes leucopenia, thrombocytopenia, and a degree of thrombocytopenia is associated with the severity of dengue fever. Therefore, the interpretation of CBC and its correlation with the particular stage of dengue fever may not be accurate in pregnancy.

Still, there are no recommendations on mode and time of delivery for mothers with dengue infection. It was only due to this mother falling critically ill, was a multidisciplinary team approach considered. As fetal maturity was satisfactory but cervix not favorable, a CS was planned under spinal anaesthesia. It was carried out as urgent procedure due to several reasons. There was a high possibility of the patient entering the critical phase within the following few hours and if it did, the persistence of severe thrombocytopenia would last for next 2–3 days, with a high risk of bleeding during that period. Therefore immediate delivery was carried out.

Miscarriages, preterm labour, fetal abnormalities, increased risk of bleeding, low birth weight and maternal death in extreme cases, have been reported as adverse effects of dengue in pregnancy [4]. There are reported cases of vertical transmission of dengue infection and they have been confirmed by identification of dengue virus in cord blood samples [5]. Although dengue virus identification in cord blood was not performed in this case, development of fever on the 5th day of life and positive NS 1 antigen without a history of mosquito bites, supported the diagnosis of vertical transmission. The onset of fever following the 4–10 days of the normal incubation period of dengue is also favors the diagnosis [7]. All reported cases of vertical transmission have occurred around the delivery, probably due to lack of sufficient time to confer the passive immunity to the fetus [5]. Duration between the onset of maternal fever and delivery affects the time of onset of dengue fever in the neonate [8]. Only a few case reports of vertical transmission of dengue are currently available and according to this limited literature, the time of onset of fever in neonates, ranged from 16 h to 11 days after birth and lasted from 2 to 6 days [8].

In conclusion, it seems that, dengue infection during pregnancy can have various adverse effects on the mother as well as on the neonate. A high index of suspicion, early diagnosis, close monitoring, timely intervention and critical consideration of physiological changes of pregnancy when interpreting clinical situation, would have led us to achieve the successful outcome of this case.

## Abbreviations

2DECHO: Two-dimensional echocardiogram
ALT: Alanine transaminase
AST: Aspartate transaminase
BP: Blood pressure
CBC: Complete blood count
CP: Critical phase
CRFT: Capillary refill time
CRP: C reactive protein
Hb: Hemoglobin
IV: Intravenous
LSCS: Lower segment caesarian section
NS-1: Non-structural protein 1
PCV: Packed cell volume
PLT: Platelet count
POG: Period of gestation
PR: Pulse rate
SBU: Special baby unit
USS: Ultrasound scan
WBC: White blood cell count
